# Extended-Interval Immunotherapy in Advanced Lung Cancer: A Case Report

**DOI:** 10.7759/cureus.104878

**Published:** 2026-03-08

**Authors:** Salome Kordzaia, Lasha Khmaladze, Elene Dolmazashvili, Tamta Makharadze, Dina Karami

**Affiliations:** 1 Medical Oncology, Multiprofile Clinic Consilium Medulla Ltd, Tbilisi, GEO; 2 Health Sciences, The University of Georgia, Tbilisi, GEO

**Keywords:** immune-related adverse event (irae), immunotherapy, lung adenocarcinoma, modified dose immunotherapy, recist 1.1

## Abstract

Lung adenocarcinoma frequently presents at an advanced stage with metastases, including the brain. Advances in immunotherapy have improved survival rates; however, challenges remain regarding optimal dosing schedules and the management of immune-related adverse events (irAEs). This case report aims to demonstrate that a less frequent immunotherapy regimen, administered every six weeks, in certain clinical scenarios may maintain durable disease stabilization while potentially reducing clinically significant toxicities. These findings support further evaluation of modified-dose immunotherapy regimens in clinical trials. We retrospectively reviewed the clinical course of a patient with advanced lung adenocarcinoma with brain and contralateral lung involvement over a 14-month period. The patient underwent biopsy confirmation, immunotherapy with atezolizumab, stereotactic radiosurgery for oligometastatic brain lesions, and subsequent therapeutic adjustments, including antibiotic treatment for pneumonia and immunotherapy regimen modifications to manage irAEs. The patient ultimately received a total of nine cycles of atezolizumab. Initial atezolizumab treatment (administered every three weeks) achieved disease stabilization (RECIST 1.1: -26.4%). The immunotherapy interval was adjusted to every six weeks during the treatment course to mitigate toxicities. Disease control was maintained with manageable irAEs, improving the patient’s quality of life. This case supports the hypothesis that, in individualized cases, a six-week immunotherapy schedule could provide durable responses in advanced lung adenocarcinoma with fewer life-threatening irAEs. These findings could inform future trials to assess the broader applicability of extended immunotherapy regimens.

## Introduction

Advanced non-small cell lung cancer (NSCLC) remains one of the leading causes of cancer-related mortality worldwide, accounting for the majority of lung cancer deaths [1]. Lung adenocarcinoma represents the most common histologic subtype of NSCLC [2]. Despite advances in screening and early detection strategies, a substantial proportion of patients present with metastatic disease at diagnosis, frequently involving the brain. Historically, prognosis in advanced NSCLC was poor; however, the advent of immune checkpoint inhibitors (ICIs) targeting the programmed death-1 and programmed death-ligand 1 (PD-L1) axes has significantly improved survival outcomes and reshaped the therapeutic landscape [3,4].

Atezolizumab, a humanized monoclonal antibody targeting PD-L1, has demonstrated clinical efficacy both as monotherapy and in combination regimens across multiple pivotal trials in advanced NSCLC [5]. Standard dosing was initially established at 1200 mg administered intravenously every three weeks [6]. Nevertheless, ICIs are characterized by prolonged half-lives and sustained receptor occupancy, leading to durable pharmacodynamic effects. Population pharmacokinetic and exposure-response modeling studies have suggested that alternative dosing regimens may achieve comparable systemic exposure and biological activity, thereby supporting extended-interval administration strategies [7,8].

Despite their therapeutic benefit, ICIs are associated with immune-related adverse events (irAEs), which may involve multiple organ systems and range in severity from mild to life-threatening [9,10]. Cutaneous toxicities are among the most frequently reported irAEs, while immune-related pneumonitis remains one of the most clinically significant and potentially serious complications in patients receiving ICI therapy [11]. Management typically includes treatment interruption and systemic corticosteroid therapy for moderate to severe cases [12]. However, optimal strategies for immunotherapy rechallenge, dose adjustment, or schedule modification following irAEs remain insufficiently defined, and evidence is largely derived from retrospective analyses [13].

In recent years, interest has grown regarding extended-interval immunotherapy dosing as a strategy to reduce treatment burden, improve quality of life, and potentially mitigate cumulative toxicity without compromising efficacy. Regulatory modeling studies have supported alternative dosing intervals for several checkpoint inhibitors, including atezolizumab [8,14]. However, real-world data evaluating intentional modification of dosing frequency following immune-related toxicity remain limited [15]. In clinical practice, treatment interruption, dose delay, or schedule modification is sometimes required in patients experiencing immune-related toxicities; however, the clinical outcomes associated with intentional modification of dosing intervals remain insufficiently characterized. Moreover, the implications of extended-interval dosing for intracranial disease control in patients with brain metastases are not fully understood, given the unique biological and pharmacokinetic considerations of CNS involvement [16,17].

Here, we present a case of stage IV lung adenocarcinoma with brain metastases managed for more than one year with atezolizumab, stereotactic radiosurgery, and supportive interventions. This case highlights the potential feasibility of modifying the immunotherapy dosing schedule to a six-week interval in a carefully selected patient who experienced immune-related toxicities while maintaining prolonged disease control. These observations underscore the need for prospective evaluation of individualized dosing strategies in advanced lung cancer.

## Case presentation

A retrospective analysis of clinical records was conducted from July 2023 to October 2024 at a tertiary care institution. This case report details a 77-year-old male diagnosed with advanced lung adenocarcinoma and active hepatitis C infection. His medical history was significant for the presence of a cardiac pacemaker and an Eastern Cooperative Oncology Group performance status of 1.

**Figure 1:**
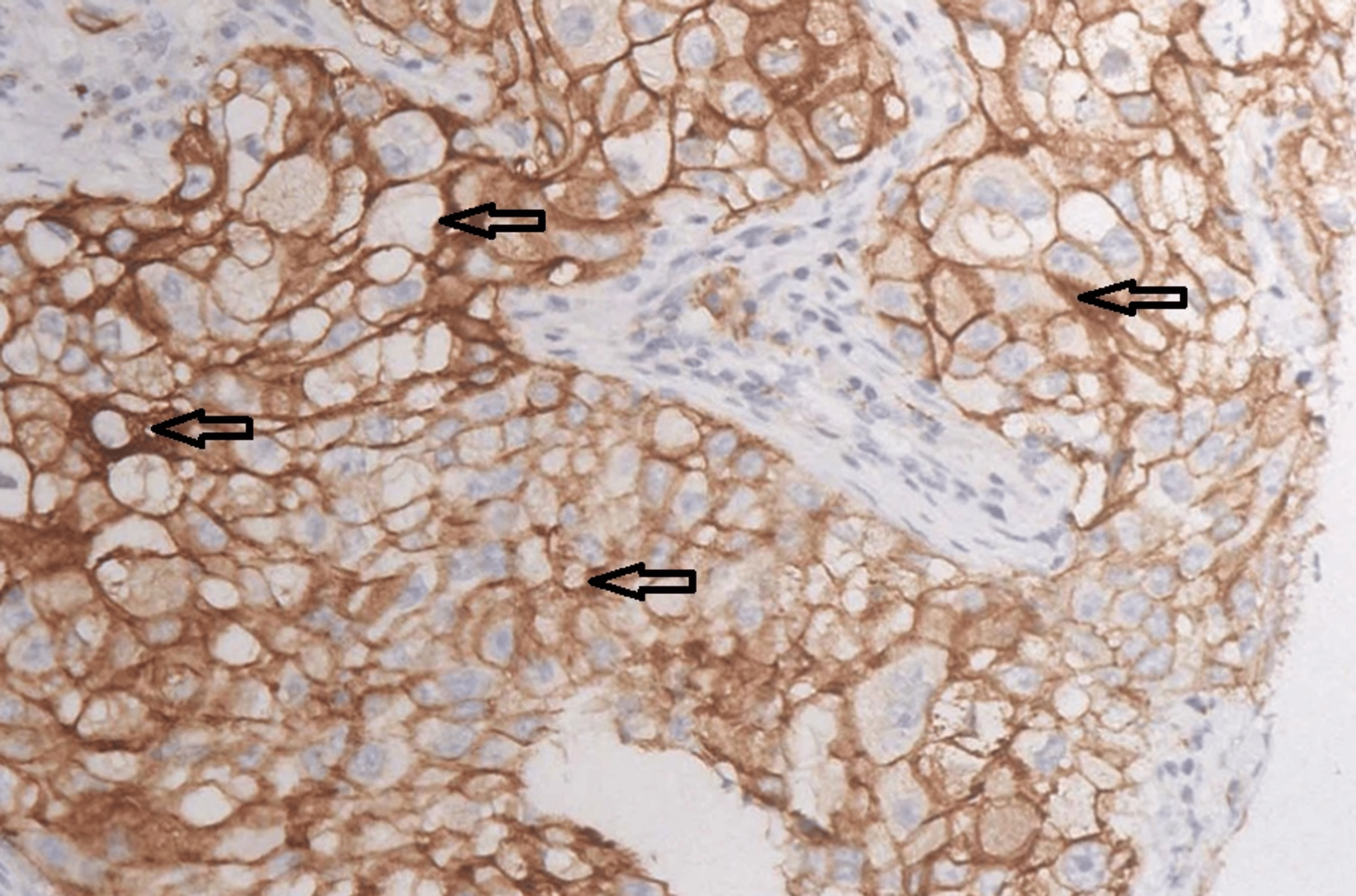
PD-L1 immunohistochemistry on biopsy specimen Biopsy specimen showing PD-L1 expression of 95%. PD-L1, programmed death-ligand 1

A lung biopsy confirmed the diagnosis of poorly differentiated (G3) adenocarcinoma, with 95% PD-L1 expression (Figure 1). No actionable driver mutations, including EGFR, ALK, or ROS1, were detected.

**Figure 2:**
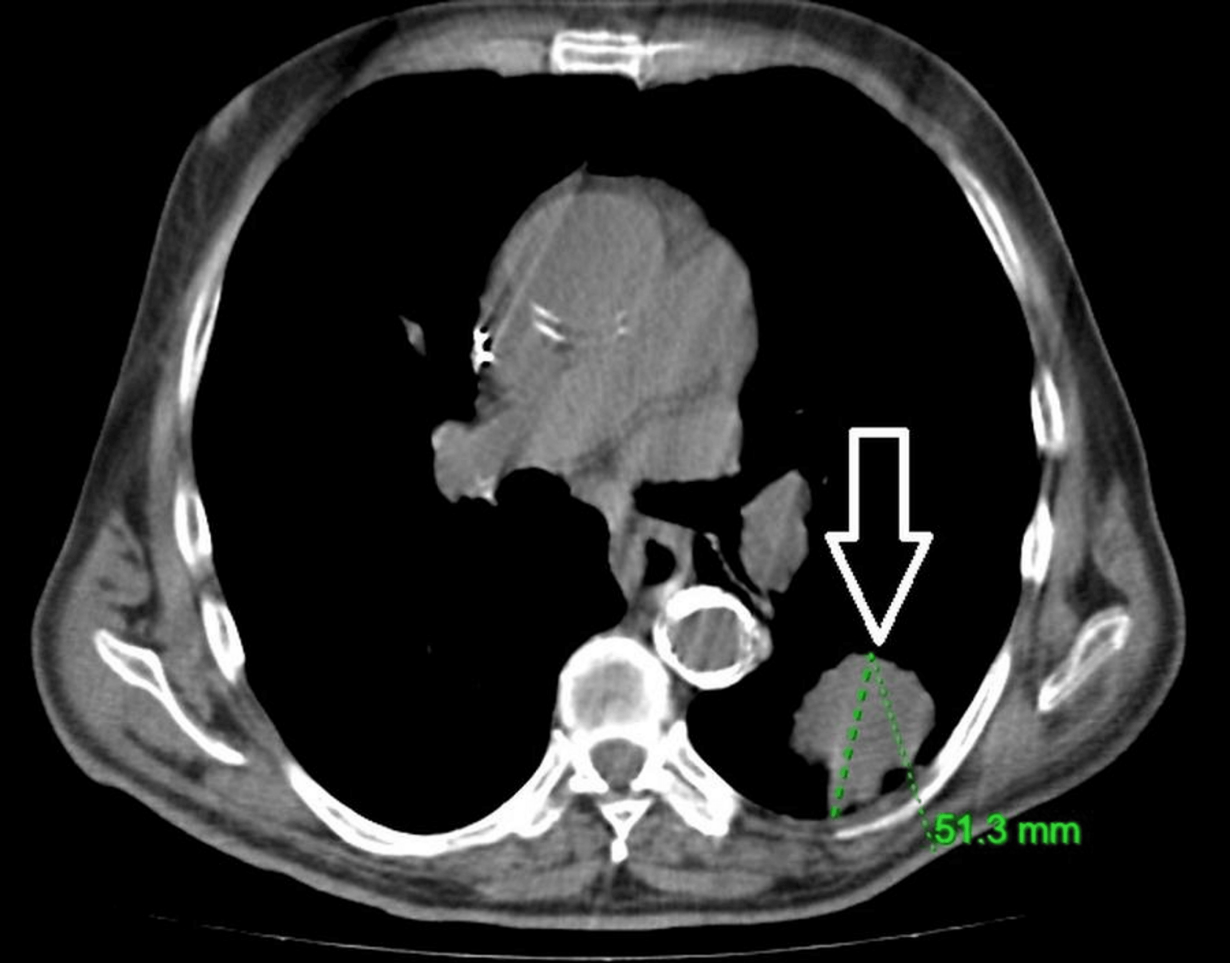
Baseline CT scan CT scan depicting the baseline target lesion measuring 5.1 cm.

**Figure 3:**
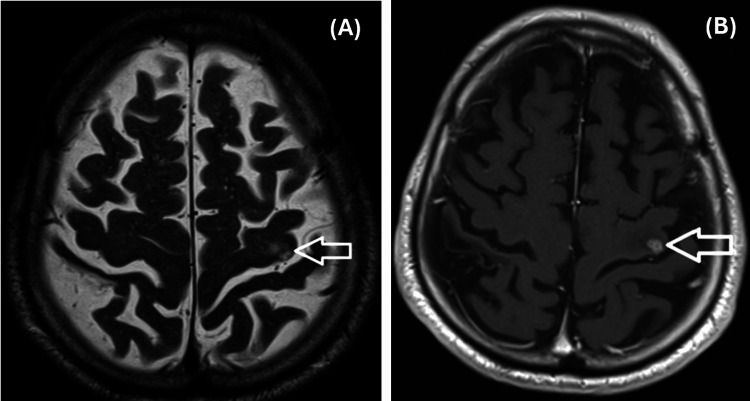
Baseline and post-SBRT MRI scans of the head (A) Baseline MRI scan depicting the solitary metastatic lesion in the left parietal lobe. (B) Post-SBRT scan depicting the solitary metastatic lesion in the left parietal lobe. SBRT, stereotactic body radiotherapy

Contrast-enhanced chest CT demonstrated a primary lesion in the left lung measuring 5.1 cm (Figure 2), with metastatic involvement of the contralateral lung. Mediastinal lymphadenopathy was also present. MRI of the brain revealed a solitary intracranial metastasis (Figure 3A), consistent with cT3N2M1b, stage IV disease.

The patient was diagnosed with active hepatitis C virus (HCV) infection. To reduce the viral load and the risk of treatment-related complications, a three-week course of antiviral therapy with a sofosbuvir-velpatasvir combination was initiated prior to the start of immunotherapy.

Initial treatment included stereotactic body radiotherapy (SBRT) to the brain (Figure 3B), followed by systemic therapy with atezolizumab at a dose of 1200 mg administered intravenously every three weeks. SBRT was re-employed during episodes of oligoprogression in the brain.

Treatment response was evaluated using the RECIST 1.1 criteria for measurable lesions. irAEs were graded according to the Common Terminology Criteria for Adverse Events (CTCAE v5.0) guidelines [18].

**Figure 4:**
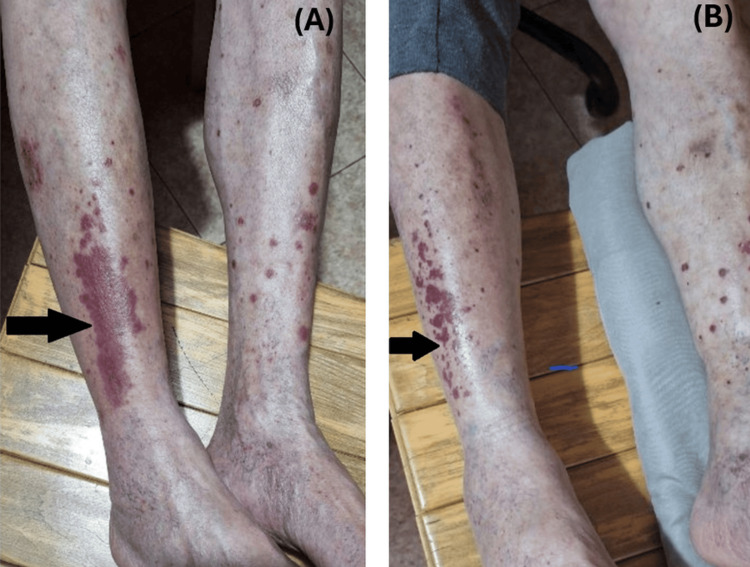
Vasculitis G1 (A) Initial presentation of grade 1 vasculitis, lower-extremity palpable purpura, non-blanching, reddish-purple macules. (B) After supportive care, the intensity and coverage area of vasculitis decreased. Image shows the resolving phase of acute vasculitis.

Five days after the first infusion, the patient developed cutaneous small-vessel vasculitis on both lower extremities (Figure 4A, 4B). The etiology remained unclear, with potential causes including HCV-associated cryoglobulinemia or an irAE induced by atezolizumab [19,20]. Given concerns that early systemic steroid use might reduce immunotherapy efficacy and compromise survival benefits, no systemic steroids were initiated [21]. Supportive measures were provided, including daily application of Betadine solution (povidone-iodine), oral administration of Ascorutin (ascorbic acid and rutoside) once daily, and topical application of Lioton gel (heparin sodium). The patient proceeded to receive the second and third cycles of atezolizumab.

**Figure 5:**
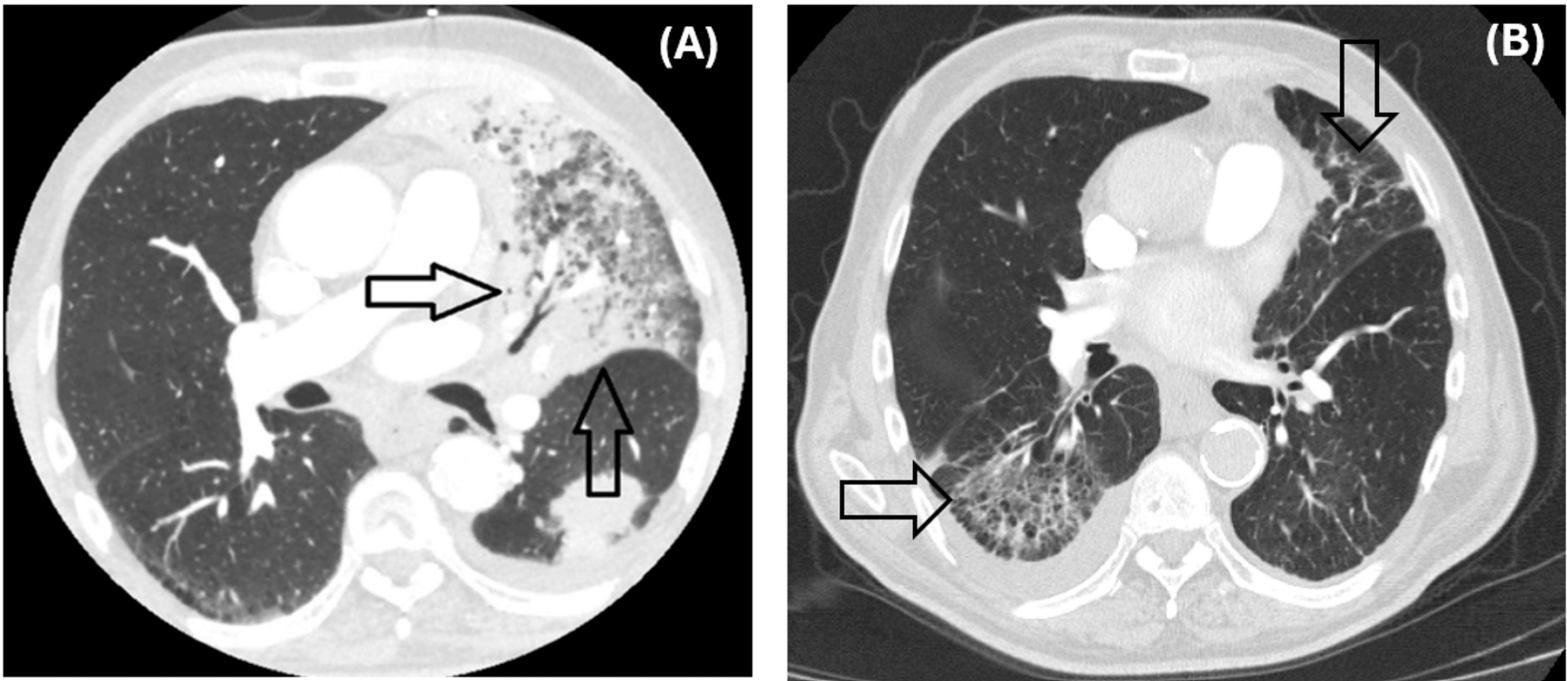
Pneumonia G2 and pneumonitis G1 (A) After three cycles of treatment, CT scan revealed left upper lobe consolidative pneumonia. The patient had shortness of breath and decreased SpO₂ (87%). Diagnosis: pneumonia grade 2. (B) After hospitalization and treatment with antibiotics, the consolidative pneumonia resolved. CT scan showed pneumonitis. The patient was asymptomatic at that time. Diagnosis: immunotherapy-induced pneumonitis grade 1.

After the third cycle, the patient developed grade 2 pneumonia (Figure 5A), which required hospitalization and treatment with intravenous antibiotics and systemic steroids. Following clinical improvement, a follow-up CT scan revealed pneumonitis (Figure 5B) graded as grade 1 due to the absence of residual symptoms. To support recovery and minimize further adverse events, the patient was started on daily vitamin D3 supplementation at a dose of 4000 IU, based on emerging evidence linking sufficient vitamin D levels to reduced severity of irAEs in patients receiving ICIs [22].

**Figure 6:**
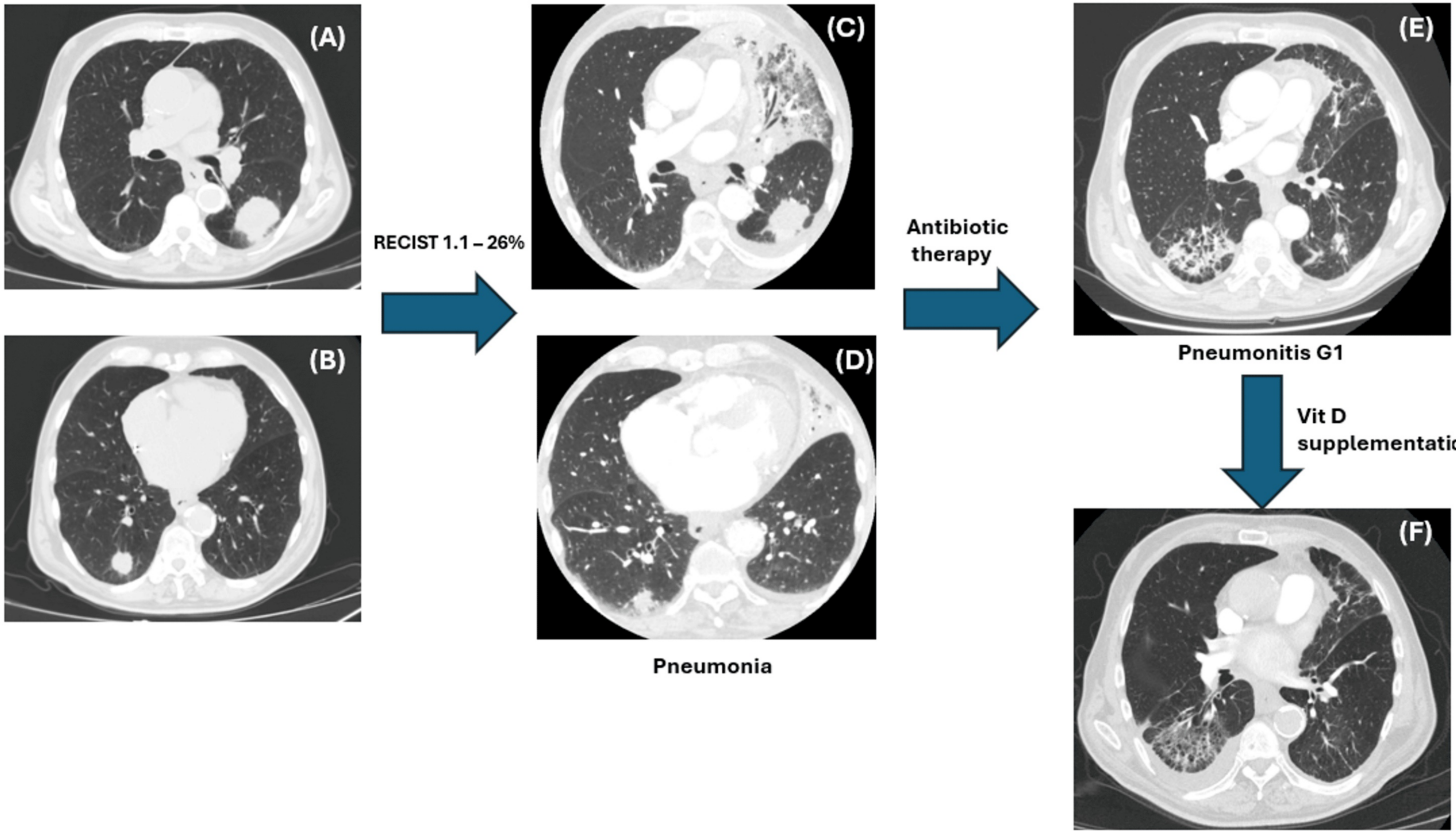
Disease stabilization and pulmonary adverse event dynamics during extended-interval immunotherapy Disease stabilization was achieved per RECIST 1.1 criteria (-26.4%) with decreasing intensity of pneumonitis (Grade 1) following vitamin D supplementation. (A, B) CT scans after three cycles of treatment demonstrating disease stabilization according to RECIST 1.1 (-26.4% from baseline). (C, D) Pneumonia phase, resolved with antibiotic therapy. (E) Pneumonitis grade 1, assessed as immunotherapy-associated. (F) Pneumonia grade 1 with positive dynamics following vitamin D supplementation.

The immunotherapy schedule was subsequently modified to a 1200 mg intravenous infusion every six weeks. Follow-up imaging showed a partial response, with a 26.4% reduction in target lesions according to RECIST 1.1. Follow-up CT scans revealed a decrease in both the area and intensity of pneumonia (Figure 6A-6F). 

**Figure 7:**
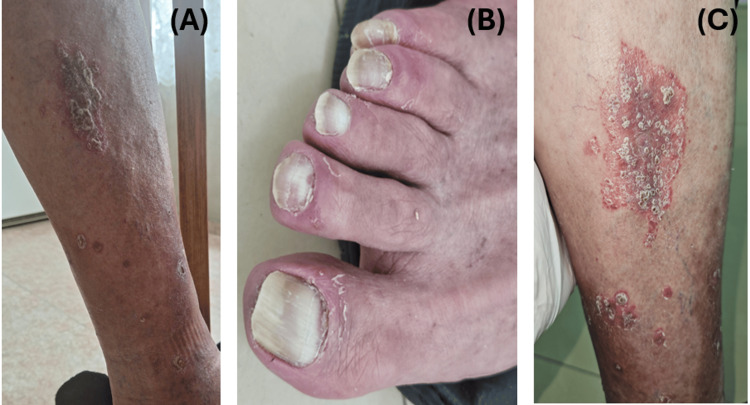
Psoriasis (A) Psoriatic plaque on the anterolateral shin of the right leg. (B) Psoriatic changes of the nails. (C) Psoriatic lesion on the medial surface of the right leg.

After the fourth cycle of immunotherapy, administered every six weeks according to the modified schedule, the patient developed grade 1 immune-related psoriasis (Figure 7A-7C). Topical therapy with Elokom (momethasone) and emollients was used, enabling the patient to continue immunotherapy without systemic corticosteroids.

The patient required SBRT to treat a 2 mm metastatic brain lesion after seven cycles of immunotherapy. Although multiple irAEs occurred, the treatment was generally well tolerated. The patient ultimately completed a total of nine cycles of atezolizumab before multiple new brain metastases were detected on follow-up brain MRI.

## Discussion

Extended-interval dosing in immunotherapy

Atezolizumab, an anti-PD-L1 ICI, has a long half-life (approximately 27 days), allowing for flexible dosing strategies [7]. Modeling studies and regulatory evaluations have suggested that longer dosing intervals, such as every six weeks, can achieve pharmacokinetic and pharmacodynamic profiles comparable to the standard three-week schedule [8].

Clinical trials such as IMpower130 and IMpower150, while not specifically designed to compare three-week and six-week intervals, provided pharmacokinetic data supporting extended dosing approvals [23]. Additionally, retrospective analyses have suggested that extended schedules can reduce healthcare burdens and improve treatment adherence, particularly among elderly patients or those with significant comorbidities [24].

Atezolizumab, like other checkpoint inhibitors, exhibits serum-dependent binding kinetics, particularly elevated dissociation rates, that may affect its pharmacologic persistence. These properties could underlie the long-lasting clinical benefit observed in our patient following transition to a six-week dosing schedule [25].

Psoriasis as a cutaneous irAE with possible prognostic implications

Several low-grade cutaneous irAEs, such as rash, pruritus, and vitiligo, have been associated with improved clinical outcomes in patients treated with ICIs [26]. Although psoriasis was not specifically reported in this meta-analysis, it shares a similar immune-mediated pathogenesis and cutaneous presentation [27]. A systematic review of 242 cases of ICI-induced psoriasis found that over half were de novo, with most patients managing symptoms without stopping treatment [28]. Therefore, the development of grade 1 immune-related psoriasis in our patient may reflect a comparable state of immune activation and could potentially carry prognostic significance.

This observation highlights the need for further investigation into the role of psoriasis as a potential marker of treatment efficacy. Future studies may help clarify the prognostic value of psoriasis in the context of ICI therapy and contribute to refining the understanding of dermatologic irAEs as predictive biomarkers.

Role of vitamin D in irAE management

Recent studies have highlighted vitamin D as a potential protective factor in the management of irAEs. Vitamin D deficiency has been associated with an increased risk of irAEs, and supplementation may offer an immunomodulatory benefit in patients receiving ICIs [29]. In our case, at the time of pneumonia and pneumonitis, the patient’s vitamin D3 level was measured at 5 ng/mL, well below the sufficient range. Daily supplementation with 4000 IU vitamin D3 successfully corrected this deficiency. Although a direct causal link cannot be confirmed, vitamin D’s immunomodulatory properties may have contributed to the observed reduction in pneumonitis severity. In addition, emerging preclinical data suggest that vitamin D may enhance antitumor immunity and improve responses to immunotherapy via microbiome-regulated pathways [16].

Efficacy of modified immunotherapy schedules

In our patient, adjusting the atezolizumab schedule to every six weeks appeared to maintain disease control while potentially minimizing toxicities. Imaging demonstrated a partial tumor response (RECIST 1.1: -26.4%) and sustained disease stabilization over nine cycles.

The adoption of a six-week dosing schedule has important implications beyond clinical efficacy. Extended-interval immunotherapy has the potential to significantly decrease hospital visit rates, improve patient convenience, and optimize resource utilization. In rural or underserved regions with limited access to oncology centers, less frequent dosing may improve treatment adherence and extend immunotherapy availability to populations historically underrepresented in clinical trials [30].

Potential inferiority of reduced-dose administration in managing CNS metastasis

Despite sustained systemic disease control in our patient, intracranial progression ultimately occurred, raising the question of whether the modified six-week dosing schedule of atezolizumab may have been suboptimal for long-term control of CNS disease. Although ICIs demonstrate clinical activity against brain metastases in NSCLC, their efficacy in the CNS remains variable and may be influenced by pharmacokinetic and biologic factors distinct from extracranial disease [31].

Atezolizumab is a large monoclonal antibody with inherently limited blood-brain barrier (BBB) penetrance under physiological conditions [32]. CNS activity is thought to occur predominantly through indirect immune-mediated mechanisms rather than direct drug exposure within the brain parenchyma. While disruption of the BBB by metastatic lesions or prior radiotherapy may facilitate immune cell trafficking, sustained systemic immune activation may be necessary to maintain intracranial disease control [33]. Prolonged dosing intervals could theoretically result in lower trough serum concentrations, potentially diminishing continuous immune pressure within the CNS.

In this context, the emergence of multiple new brain metastases despite controlled pulmonary disease suggests a possible compartmental dissociation between systemic and intracranial responses. Although causality cannot be established in a single case, this observation raises the hypothesis that extended-interval immunotherapy may be less effective for CNS disease control in certain patients. These findings highlight the need for caution when modifying immunotherapy schedules in patients with known or high-risk brain metastases and underscore the importance of dedicated prospective studies evaluating CNS outcomes under reduced-frequency or alternative dosing regimens.

## Conclusions

This case report illustrates the feasibility of an extended six-week dosing interval of atezolizumab in a carefully selected patient with advanced lung adenocarcinoma who had multiple comorbidities and experienced treatment-related complications. Despite these challenges, the patient completed nine cycles of therapy with only low-grade toxicities after dose adjustment, which did not interfere with treatment continuation. The extended interval not only facilitated durable disease control but also maintained irAE occurrence within acceptable limits, rendering them manageable and preserving the patient’s quality of life throughout treatment.

While this report provides valuable insights, the findings are based on a single case. Consequently, future research should investigate the role of predictive biomarkers, such as PD-L1 expression and baseline vitamin D levels, in optimizing patient selection for extended immunotherapy dosing schedules. Well-designed prospective trials comparing standard (every three weeks) and extended (every six weeks) dosing schedules are essential to validate the safety, efficacy, and cost-effectiveness of this strategy across diverse patient populations.
